# In Situ Grafted Composite Nanoparticles-Reinforced Polyurethane Elastomer Composites with Excellent Continuous Anti-Impact Performance

**DOI:** 10.3390/ma14206195

**Published:** 2021-10-19

**Authors:** Feng Qi, Zhuoyu Zheng, Zehui Xiang, Biao Zhang, Fugang Qi, Nie Zhao, Xiaoping Ouyang

**Affiliations:** 1 School of Materials Science and Engineering, Xiangtan University, Xiangtan 411105, China; 201931000115@smail.xtu.edu.cn (F.Q.); 202021001772@smail.xtu.edu.cn (Z.Z.); zhaonie@xtu.edu.cn (N.Z.); oyxp2003@aliyun.com (X.O.); 2 Qingdao Green World New Material Technology Co., Ltd., Qingdao 266100, China; 3 Key Laboratory of Low Dimensional Materials and Application Technology of Ministry of Education, Xiangtan University, Xiangtan 411105, China

**Keywords:** polyurethane elastomer, composite nanoparticles, continuous impact resistance

## Abstract

Polyurethane elastomer (PUE) has attracted much attention in impact energy absorption due to its impressive toughness and easy processability. However, the lack of continuous impact resistance limits its wider application. Here, an amino-siloxane (APTES) grafted WS_2_-coated MWCNTs (A-WS_2_@MWCNTs) filler was synthesized, and A-WS_2_@MWCNTs/PUE was prepared by using the filler. Mechanical tests and impact damage characterization of pure PUE and composite PUE were carried out systematically. Compared with pure PUE, the static compressive strength and dynamic yield stress of A-WS_2_@MWCNTs/PUE are increased by 144.2% and 331.7%, respectively. A-WS_2_@MWCNTs/PUE remains intact after 10 consecutive impacts, while the pure PUE appears serious damage after only a one-time impact. The improvement of mechanical properties of A-WS_2_@MWCNTs/PUE lies in the interfacial interaction and synergy of composite fillers. Microscopic morphology observation and damage analysis show that the composite nanofiller has suitable interfacial compatibility with the PUE matrix and can inhibit crack growth and expansion. Therefore, this experiment provides an experimental and theoretical basis for the preparation of PUE with excellent impact resistance, which will help PUE to be more widely used in the protection field.

## 1. Introduction

Polyurethane elastomer (PUE), as a well-know (AB)n-type block linear polymer, has attracted much attention recently from industry and academia due to its light weight, high strength, and suitable recovery performance applications [1,2]. However, the problems such as low impact resistance, especially continuous impact, restrict its application as a high-performance impact protection material. In impact applications, PUE usually needs to resist impact and shear forces, which requires PUE to have excellent toughness and strength. The hard segments of PUE serve as tougheners, dissipating energy during deformation, resulting in a high toughness [3]. Nevertheless, the toughness obtained by energy dissipation is ineffective in resisting crack growth under cyclic impact. At this time, the strength of PUE plays a decisive role in hindering crack formation and propagation [4]. Due to the synergistic effect between nanofillers and polymer matrix, incorporating nanoparticles into polymers is an effective way to develop excellent polymer composites with strength and toughness [5].

Nanoparticles-reinforced polymer composites have gradually received more and more attention due to their specific and attractive mechanical properties and thus have unique applications in impact protection sectors [6]. Therefore, nanomaterials with high intensity and excellent energy absorption characteristics, such as multi-walled carbon nanotubes (MWCNTs), can be used as effective impact-resistant fillers to improve the mechanical properties of the polymer [7,8]. However, the dispersibility and interface compatibility of MWCNTs and polymer matrix are not ideal, and the nanoparticle-polymer interface appears to be one of the important to realize the enhancement of mechanical properties of polymer composite materials [9]. Two or more nanoparticles are added simultaneously to the polymer system is an alternative way for enhancing filler dispersion and interfacial interaction without using other traditional approaches [10]. According to the research of Gao et al. [11], the mobility of interfacial polymer beads is the slowest for the nanosheet-filled system. Only in this case will there be a strong, attractive interfacial interaction between the nanoparticle and the polymer matrix. In addition, Liu et al. [12] studied the interface interactions of graphene, C_60,_ and CNT with polymers. It was found that compared with CNT and C_60_, graphene sheets had stronger interface interaction and larger surface area, which greatly improved the occurrence of fracture. Hence, under the premise of retaining the excellent mechanical properties of MWCNTs, wrapping nanosheets on the surface of MWCNTs can enhance the interface interaction between nanoparticles and polymers.

Tungsten disulfide (WS_2_) is a member of the transition metal disulfide (TMD) family and has a layered structure similar to graphene [13]. Some studies have demonstrated that WS_2_ nanosheets are solid lubricants that enhance the toughness of polymers and have strong interface interactions with the polymer matrix. Therefore, the coating of MWCNTs by WS_2_ nanosheets can effectively enhance the interface interaction between MWCNTs and the polymer matrix and promote the improvement of the impact resistance of the nanofiller-polymer system. In addition, the use of silane coupling agents to improve the agglomeration of nanofiller is a common and effective method [14]. Zhang et al. [15] added inorganic fullerene-like (IF) WS_2_ nanoparticles treated by a coupling agent to the precursor solution of UHMWPE. IF-WS_2_ nanoparticles modified by silane coupling agent significantly improved the bulletproof performance of UHMWPE fiber. Divya et al. [16] used silane coupling agent for surface treatment of SiO_2_. The dispersion of SiO_2_ in epoxy matrix was improved, and the surface hardness, tensile properties, and bending properties of epoxy resin were improved. Kim et al. [17] modified BN/Fe_3_O_4_ composite nanoparticles with silane coupling agent, which made the nanoparticles enhance the thermodynamic properties of the polymer. Therefore, selecting an appropriate silane coupling agent can further improve the dispersion and interfacial interaction of nanofillers.

In this paper, multi-walled carbon nanotubes are used creatively to prepare WS_2_ nanosheet-coated multi-walled carbon nanotubes composite nanoparticles (WS_2_@MWCNTs) by in situ growth method. Silane coupling agents (γ-Aminopropyl triethoxysilane) containing amino functional groups are used for in situ grafting on the surface of the composite nanoparticles (A-WS_2_@MWCNTs). The suitable interface interaction between A-WS_2_@MWCNTs and PUE matrix greatly improves the mechanical properties of PUE. More importantly, the results of the continuous impact test show that the composite PUE added with A-WS_2_@MWCNTs has excellent continuous anti-impact cycle service performance and energy absorption characteristics. In addition, an optical microscope, scanning electron microscope, and ultra-depth of field three-dimensional microscopy system are used to characterize the sample, explore the damage mechanism of the sample, and explain the role of A-WS_2_@MWCNTs in the impact process. This research provides an effective solution for the preparation and improvement of nanocomposite PUE with continuous impact resistance.

## 2. Materials and Methods

### 2.1. Materials

All PUE materials were supplied by Qingdao Green World New Material Technology Co., Ltd. (Qingdao, China), which include two components: A (a toluene diisocyanate (TDI): –NCO, 6.212 wt.%) and B (a polyol: –OH, 6.358 wt.% and American Vertellus Coscat AC-83 Organobismuth catalyst, 0.3 wt.%). Other related raw materials including ethyl thioacetamide (C_2_H_5_NS, AR, 99%), tungsten chloride (WCl_6_, 99%), multi-walled carbon nanotubes (MWCNTs, ≥95%, ID: 5–12 nm, OD: 30–50 nm, length: 10–20 µm), γ-aminopropyl triethoxy silane (APTES) reagents were purchased from Shanghai Macklin Biochemical Technology Co., Ltd (Shanghai, China).

### 2.2. Preparation of Composite Materials

**Figure 1:**
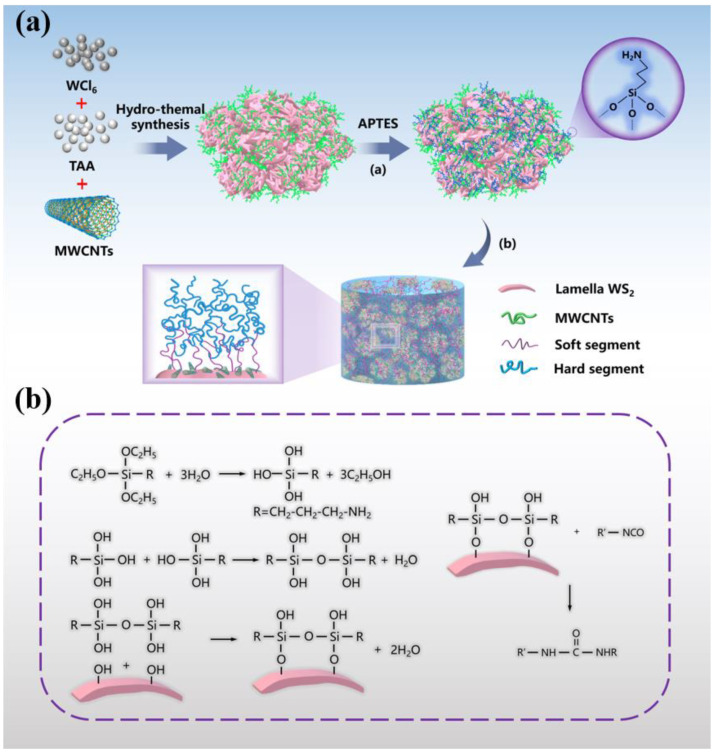
(**a**) Preparation of WS_2_@MWCNTs composite nanoparticles by in situ growth method, and (**b**) chemical schematic diagram of A-WS_2_@MWCNTs prepared by in situ grafting.

The overall experimental process and chemical reaction are shown in Figure 1. The raw materials of component A and component B were mixed in the weight ratio of 5:2 and then poured into the mold to remove bubbles. Pure PUE was made by compression molding.

#### 2.2.1. Preparation of WS_2_@MWCNTs Composite Nanoparticles

The synthesis method of composite nanoparticles (WS_2_@MWCNTs) was as follows: C_2_H_5_NS (3.2 g) was poured into 10 mL ultrapure water for magnetic stirring for 30 min, and then WCl_6_ (4.45 g) was added into the dispersion solution for further reaction for 1 h. Then, MWCNTs (1.85 g) were weighed into the above solution and dispersed ultrasonically for 2 h. The mixed solution was poured into the reactor and reacted at 200 °C for 24 h. After the reaction, the mixed solution was washed and filtered several times with acetic acid and ultrapure water. The filtered solid was dried at 100 °C in a vacuum environment for 24 h to obtain the product WS_2_@MWCNTs.

#### 2.2.2. Preparation of A-WS_2_@MWCNTs/PUE Composite Materials

The synthesis of APTES-WS_2_@MWCNTs (A-WS_2_@MWCNTs) was as follows: First, APTES (4 mL) was dropped into a mixed solution of ethanol (40 mL) and ultrapure water (10 mL) for magnetic stirring for 30 min. Then composite nanoparticles (1 g) were added to the solution after hydrolysis, adjust the PH between 4 and 5 with an appropriate amount of acetic acid, ultrasonicate for 2 h, and magnetic stirring at 60 °C for 6 h. After the reaction was completed, wash with ethanol and water until neutral. Finally, the product was vacuum dried at 60 °C for 12 h and then ground for later use. Finally, A-WS_2_@MWCNTs was added to B component after ultrasonic dispersion, and then A component was added, mixed evenly, and poured into the mold to form A-WS_2_@MWCNTs/PUE composite material (The cylinder model with a diameter of Φ 20 × 4 mm was used for static pressure test, and the cylinder model with diameter Φ 10 × 2 mm was used for SHPB test). The added information of nanofillers is shown in Table 1.

### 2.3. Characterization Methods

Microscopic morphology of nanoparticles and microscopic damage of specimen surface as well as cross-section after impact observed by scanning electron microscopy (SEM, Zeiss, Sigma300) (Carl Zeiss, Baden-Württemberg, Germany). The phases of the composite nanoparticles were analyzed by X-ray diffraction (XRD, Bruker, D8 Advance, Karlsruhe, Germany) at a scanning angle of 10°–80° and a scanning speed of 10°/min. The grafting of composite nanoparticles was characterized by Fourier transform infrared spectroscopy (FTIR, Nicolet 380, Waltham, MA, USA), and the powder samples were prepared by the pressing sheet method. Raman spectroscopy (Raman, Renishaw, inVia, London, UK) was used to characterize the area ratio of peak D to peak G of MWCNTs. A universal testing machine (Hua long, WDW-100C, Shanghai, China) was used for static compression tests with a compression rate of 1.2 mm/min and a compression thickness of 3.5 mm. Three parallel experiments were conducted for all samples to take the average value [18,19]. Aluminum Hopkinson Bar (made in National University of Defense Technology, Changsha, China) was used to measure the dynamic impact resistance of specimens [20,21,22,23]. An optical microscope (Olympus, BX53M, Tokyo, Japan) and ultra-depth of field 3D microscope system (Keyence, VHX-6000, Osaka, Japan) were used to observe the damaged microscopic appearance of the specimen after the impact.

## 3. Results and Discussions

### 3.1. Characterization of Composite Nanoparticles

**Figure 2:**
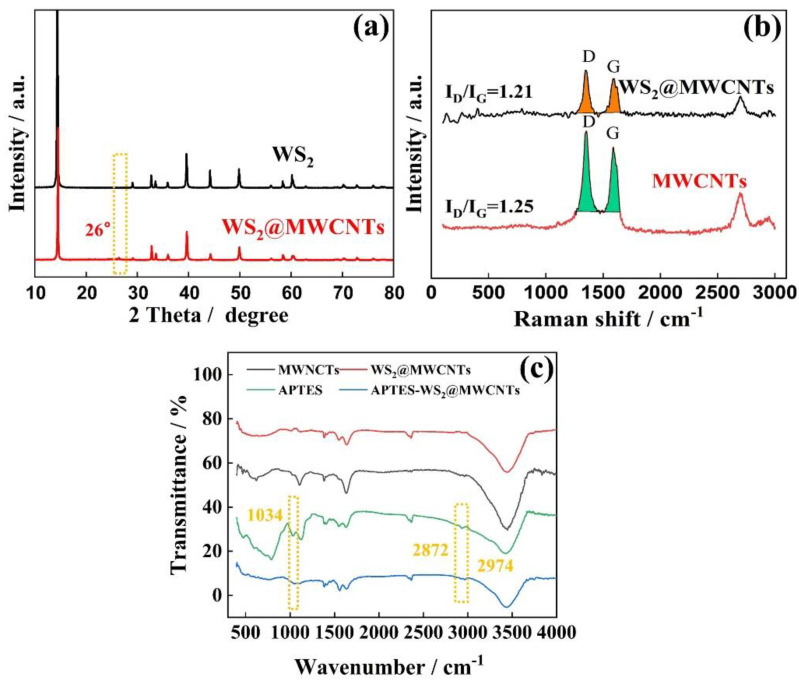
Basic characterization of nanoparticles. (**a**) XRD images of MWCNTs and WS_2_@MWCNTs, (**b**) Raman spectroscopy of WS_2_ and WS_2_@MWCNTs, and (**c**) FTIR spectroscopy of MWCNTs, WS_2_@MWCNTs, APTES, and APTES-WS_2_@MWCNTs.

As shown in Figure 2a, the crystal structure and phase purity of WS_2_@MWCNTs composite nanoparticles grown in situ are studied. The main diffraction peak observed is consistent with the main diffraction peak of hexagonal crystal system WS_2_. The diffraction peak corresponding to MWCNTs (002) is about 26° [13]. Figure 2b is the Raman spectra of MWCNTs and WS_2_@MWCNTs. MWCNTs are coated with WS_2_, which reduces the effective vibrational groups of MWCNTs. The peak strength of WS_2_@MWCNTs decreases significantly. However, the area ratio between the D peak (Peak D represents the SP^3^ hybridization of the C atom) and the G peak (Peak D represents the SP^2^ hybridization of the C atom) does not change significantly. This means that MWCNTS is coated by WS_2_ and will not destroy its own structure [24]. Figure 2c is the infrared spectrum of MWCNTs, WS_2_@MWCNTs, and A-WS_2_@MWCNTs nanoparticles. WS_2_@MWCNTs grafted with APTES have strong Si-O-Si vibration at 1034 cm^−1^, and C-H symmetric and asymmetric tensile vibration peaks at 2974 and 2872 cm^−1^.

**Figure 3:**
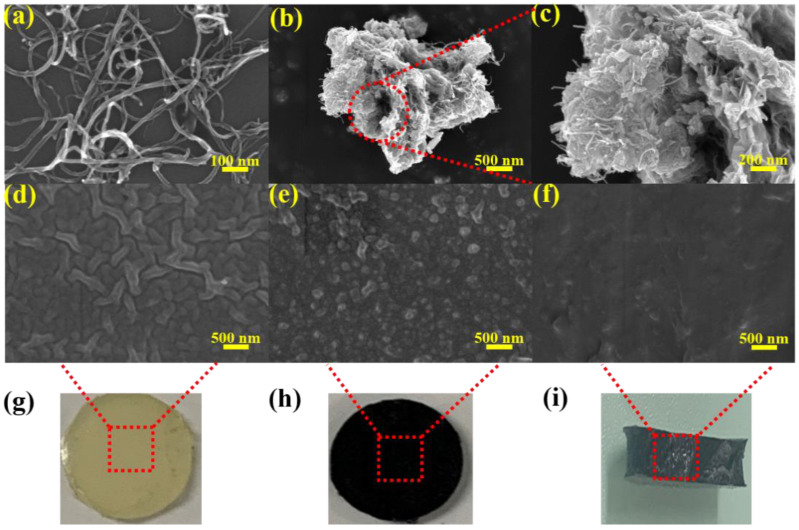
(**a**) SEM image of MWCNTs, (**b**) SEM image of WS_2_@MWCNTs, (**c**) SEM image corresponding to the selected area in (**b**), (**d**,**e**) SEM images of pure PUE and A-WS_2_@MWCNTs/PUE surface corresponding to the selected area in (**g**,**h**), and (**f**) SEM image of A-WS_2_@MWCNTs/PUE fracture corresponding to the selected area in (**i**).

Figure 3a–c shows the microstructure of MWNCTs and WS_2_@MWCNTs. The in situ grown WS_2_ nanosheets are coated with MWCNTs, which is beneficial to enhance the interface interaction between the nanofillers and the PUE matrix. The surface micro morphologies of pure PUE and 1.5%A-WS_2_@MWCNTs/PUE are shown in Figure 3d,e. Compared with the surface of pure PUE, the surface microstructure of A-WS_2_@MWCNTs/PUE is rougher with obvious protrusions. However, the A-WS_2_@MWCNTs nanofiller wrapped in a polyurethane matrix has suitable compatibility. Figure 3f shows the microstructure of 1.5% A-WS_2_@MWCNTs/PUE fracture. It can be seen that A-WS_2_@MWCNTs are uniformly distributed in the PUE matrix. It proves that the filler has suitable interface interaction with the polymer matrix and makes a great contribution to the improvement of anti-impact performance.

### 3.2. Static Mechanical Characterization

**Figure 4:**
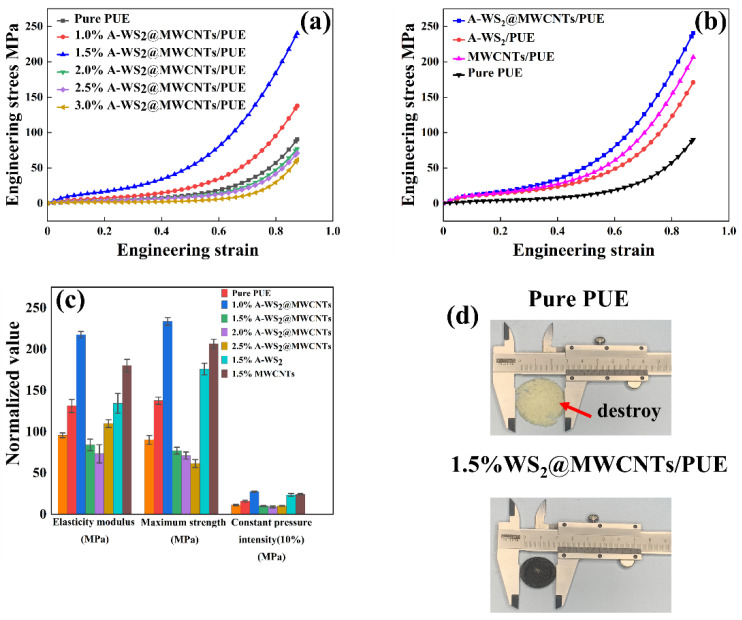
(**a**) Stress-strain curves of composite PUE with 1%–3% mass fraction A-WS_2_@MWCNTs filler, (**b**) composite PUE stress-strain curves with the addition of A-WS_2_, MWCNTs, and A-WS_2_@MWCNTs fillers (the added mass fraction was 1.5%), (**c**) values of elasticity modulus, maximum strength and constant pressure intensity corresponding to the tested samples in (**a**,**b**), and (**d**) comparison of the damage level of pure PUE and A-WS_2_@MWCNTs/PUE after compression.

Cylindrical specimens (Φ 20 × 4 mm) are selected for static compression test. As shown in Figure 4a, the addition of 1.5% A-WS_2_@MWCNTs has the best effect on improving the anti-compression performance of PUE. When the filler addition exceeds 2%, the compression resistance of the composite PUE will be severely reduced. A-WS_2_@MWCNTs fillers are usually unevenly dispersed in the polymer matrix due to their high specific surface energy, which will cause the filler to accumulate and agglomerate under higher filler loading [25]. Figure 4b shows the effect of uncompounded nanoparticles on the compression performance of PUE at 1.5% mass fraction filling. The addition of uncompounded WS_2_ and MWCNTs alone is also able to enhance the static compression performance of PUE, which can be attributed to the homogeneous dispersion of nanofillers that has an enhancing effect on the mechanical properties of the polymer [8,13,26]. However, A-WS_2_@MWCNTs is still the most obvious improvement in static compression strength. Under the same compression conditions, pure PUE has been completely destroyed, while A-WS_2_@MWCNTs/PUE remains in its original state (Figure 4d). Compared with pure PUE, the elastic modulus of A-WS_2_@MWCNTs composite PUE is increased by 127.1%, the maximum pressure is increased by 159%, and the 10% constant pressure strength is increased by 144.2% (Figure 4c and Table 1). It is speculated that the reasons for the improvement of its mechanical properties are as follows: (I) Strong interfacial interaction between A-WS_2_@MWCNTs and PUE [27]. A-WS_2_@MWCNTs composite nanoparticle system has suitable compatibility with PUE. The organic polymers and inorganic nanoparticles form a strong interface force. During the compression process, the load force is transferred from the polymer chain to the rigid nanomaterial, thereby effectively increasing the compressive strength of the composite material. (II) The enthalpy and entropy interaction of amino-functionalized WS_2_@MWCNTs in the nanoparticle-polymer composites can guide the arrangement and distribution of nanoparticles [28], and the overall density of the matrix increases, which is beneficial to the improvement of mechanical properties [27]. (III) The hydroxyl groups contained in the composite nanofiller form hydrogen bonds with the carbonyl groups of polyurethane in polyurethane. It produces physical cross-linking, which limits the movement of polyurethane molecular chains during compression and thus improves the compressive strength of polyurethane.

In particular, it should be pointed out that the influence of static compression test and dynamic impact test on the deformation of PUE lies in the difference of strain rate. As a typical strain rate material, PUE will have a strain rate effect and glass transition effect under a high strain rate, which is not available in static compression tests [29,30]. Therefore, the static compression test is different from the dynamic impact test. In order to further study the continuous anti-impact performance of the composite PUE, a series of dynamic impact tests are necessary.

### 3.3. Dynamic Impact Test

A series of dynamic impact tests of PUE at different strain rates are carried out by using a split Hopkinson pressure bar (SHPB). SHPB is mainly composed of impact rod, input rod, and output rod. All rods are made of aluminum alloy.

In the SHPB test, the size of the sample has a great influence on the final reliable data. For the softer polymer materials, a smaller ratio of length to diameter (L/D = 0.2) is beneficial to avoid the end effect and inertia effect and keep the uniform deformation and stress balance. In addition, the uniform application of Vaseline on the rod end is beneficial to reduce the friction effect on the test. Because the change of sample size with time cannot be measured accurately, and the change of sample size after impact is small, the dynamic stress, strain, and strain rate are calculated by using the initial cross-sectional area and length of the sample. According to the one-dimensional wave propagation theory, the strains recorded by the incident, transmitted, and reflected waves are used to derive the stress, strain, and strain rate over time. They have the following relationships:

(1) $$\sigma_{s}\left(t\right)=E_{0}\frac{A_{0}}{A_{s0}}\varepsilon_{t}\left(t\right),$$

(2) $$\varepsilon_{s}\left(t\right)=-\frac{2C_{0}}{L_{s0}}\int_{0}^{t}\varepsilon_{r}\left(t\right)dt,$$

(3) $${\dot{\varepsilon }}_{s}\left(t\right)=-\frac{2C_{0}}{L_{s0}}\varepsilon_{r}\left(t\right),$$

In the formula, *σ_s_*(*t*), *ε_s_*(*t*), and *ε_s_*(*t*) are the stress, strain, and strain rate of the sample under test with time; *ε_r_*(*t*) and *ε_t_*(*t*) are recorded strains with time for the input and output rods, respectively; *A_s*0*_* and *L_s*0*_* are the initial cross-sectional area and length of the tested specimen; *E*_0_ is the Young’s modulus of bars; *A*_0_ is the cross-sectional area of bars; and *C*_0_ = *ρ_E*0*_0*/*ρ*_0_ (*ρ*_0_ is the density of bars) is the wave velocity in bars.

The energy absorbed by a material under a high-speed impact is defined as the strain energy *(U*) per unit volume (*V*) and is equal to the area of the stress-strain curve measured from *ε*_0_ to *ε*_1_. It can be expressed in terms of strain energy density (*u*), which can be expressed by the following equation:

(4) $$u=\frac{U}{V}=\int_{0}^{\varepsilon_{1}}\sigma_{x}d\varepsilon_{x},$$

where *σ_x_* is the normal stress in the bar, *ε_x_* is the normal strain, and *ε*_1_ is the normal strain corresponding to elongation *x*_1_ [31,32].

#### 3.3.1. One-Time Impact Test

**Figure 5:**
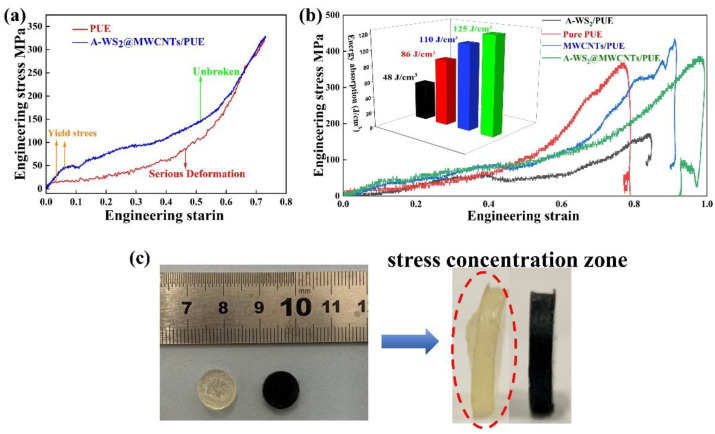
(**a**) Stress-strain curve of PUE and 1.5%A-WS_2_@MWCNTs/PUE under 0.2 MPa impact load, (**b**) stress-strain curve and energy absorption value of 1.5%A-WS_2_/PUE,1.5% MWCNTs/PUE, and 1.5%A-WS_2_@MWCNTs/PUE under the 0.2 MPa impact load, and (**c**) deformation of pure PUE and 1.5%A-WS_2_@MWCNTs/PUE under 0.2 MPa impact load.

The engineering stress-strain curves of pure PUE and A-WS_2_@MWCNTs/PUE under the impact force of 0.2 MPa are shown in Figure 5a. In the initial elastic deformation stage, the stress-strain relationship is linear, and its slope is dynamic Young’s modulus, which represents the material’s ability to resist deformation. Compared with pure PUE, the dynamic Young’s modulus of A-WS_2_@MWCNTs/PUE is increased by 167.2%. After the elastic deformation stage, a nonlinear transition occurs at the dynamic yield stress point. Then a large strain occurs under the platform stress until the material densifies, accompanied by a significant increase in stress. In this process, the material has no obvious softening phenomenon, so the dynamic yield stress is consistent with the platform stress, which is a key parameter for evaluating the material’s strain energy absorption and impact resistance [33,34,35]. Compared with pure PUE, the dynamic yield stress of A-WS_2_@MWCNTs/PUE is increased by 331.7%. Moreover, compared with the dynamic yield stress of PUE or PUA prepared by predecessors, this research also has great advantages (Present work: 51 MPa, Boyce MC’s PUE [36]: 38 MPa, Roland CM’s [37] PUA: 26 MPa, Yao X H’s PUE [38]: 20 MPa). Figure 5b shows the influence of the addition of different nanomaterials on the impact resistance of PUE. The addition of A-WS_2_@MWCNTs and MWCNTs improves energy absorption. However, the addition of A-WS_2_ reduces the energy absorption of the PUE. It is attributed to the fact that WS_2_ nanosheets only increased the toughness of PUE but not the strength of PUE. Therefore, only the addition of A-WS_2_@MWCNTs can enhance both the toughness and strength of PUE. In addition, Figure 5c shows that pure PUE suffers irreversible damage after impact. Due to the concentration of stress, the middle part has been completely destroyed. However, A-WS_2_@MWCNTs/PUE does not show any damage.

#### 3.3.2. Continuous Cycle Impact Test

**Figure 6:**
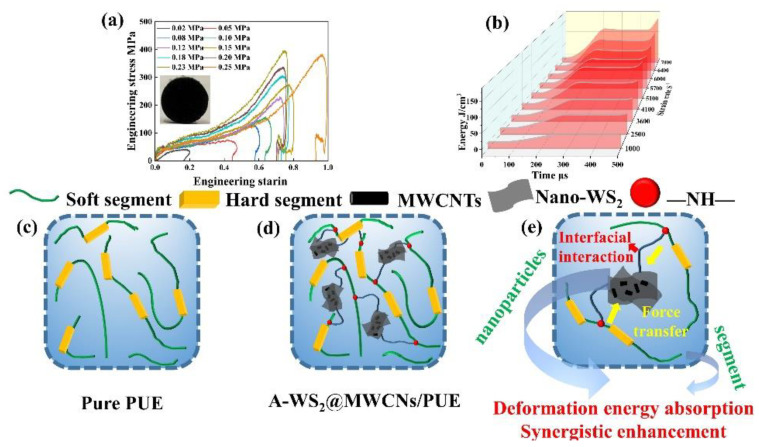
(**a**) Stress-strain curve of 1.5%A-WS_2_@MWCNTs/PUE under 10 continuous impacts, (**b**) 1.5%A-WS_2_@MWCNTs/PUE energy absorption platform under 10 continuous impacts, (**c**,**d**) schematic diagram of pure PUE and A-WS_2_@MWCNTs/PUE internal chain segments, and (**e**) schematic diagram of A-WS_2_@MWCNTs-enhanced PUE impact resistance.

Figure 6a shows the stress-strain curve (0.02–0.25 MPa) under the 10 successive impacts. As the impact load increases, the maximum stress of A-WS_2_@MWCNTs/PUE continues to rise. In the initial low-velocity impact stage, A-WS_2_@MWCNTs/PUE relies on its own high strength to resist impact. When the impact load is loaded to 0.25 MPa, it is found that the maximum stress no longer rises, but the strain rate has increased greatly. In this process, A-WS_2_@MWCNTs rely on their own suitable toughness to undergo plastic deformation to increase energy absorption (Figure 6b). The increase in toughness can be attributed to the strain rate effect and glass transition characteristics of PUE (strain rate material) [38]. Figure 6c,d illustrates the continuous impact resistance enhancement mechanism of A-WS_2_@MWCNTs. Excellent interfacial interaction between nanoparticles and the PUE matrix is a prerequisite for enhanced mechanical properties, which promotes the force transfer from the PUE matrix to the nanoparticles. In addition, energy absorption resulting from plastic deformation of chain segments, synergistic toughening between nanoparticles and PUE matrix, van der Waals forces, and synergistic effect between nanoparticles are also important reasons for the improvement of the impact resistance of A-WS_2_@MWCNTs/PUE [39].

### 3.4. Craze Evolution, Crack Formation, and Destruction Mechanism

The OM, SEM, and ultra-depth of field 3D microscope system are used to observe and analyze the surface, cross-section, and overall morphology of the sample.

**Figure 7:**
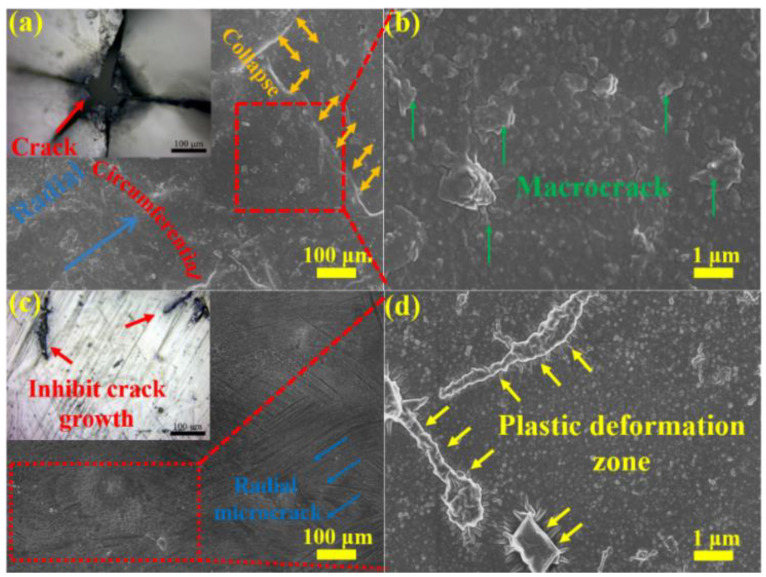
(**a**,**c**) Optical and SEM microscopes images of (**a**) pure PUE and (**c**) A-WS_2_@MWCNTs/PUE surface, and (**b**,**d**) SEM images corresponding to the selected area in (**a**,**c**).

Figure 7 shows the surface micro morphologies of pure PUE and A-WS_2_@MWCNTs/PUE after impact. Figure 7a,b is the surface morphologies of pure PUE. It can be seen that the middle part of the pure PUE has suffered serious perforation damage. The reason is that the shear band formed under the combined action of the circumferential stress and the radial stress moves to the center, causing stress concentration damage. However, as shown in Figure 7c,d, there is no concentrated shear band in the center of A-WS_2_@MWCNTs/PUE, and a few cracks on the surface do not continue to expand in the radial direction. Under SEM observation, it is found that there is no macroscopic continuous expansion of collapse and spalling, but a partial plastic deformation zone appears. This plastic zone retards the expansion of macroscopic cracks and improves the impact toughness.

**Figure 8:**
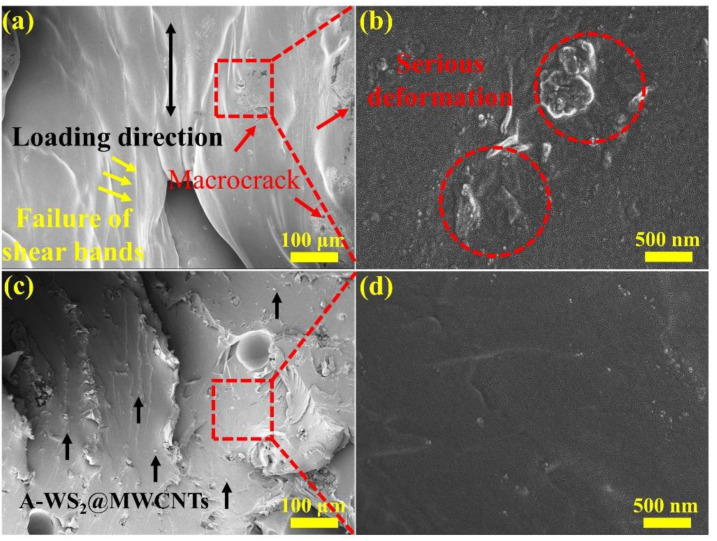
(**a**,**c**) SEM images of (**a**) pure PUE and (**c**) A-WS_2_@MWCNTs/PUE fracture, and (**b**,**d**) SEM images corresponding to the selected area in (**a**,**c**).

Figure 8 shows SEM images of the cryo-fracture surface of the pure PUE samples and the A-WS_2_@MWCNTs/PUE samples after impact. Figure 8a,b shows the shear failure zone and part of the spalled fragments in the direction of pure PUE impact loading. This proves that pure PUE has been completely destroyed under impact loading. On the contrary, as follows from these SEM images (Figure 8c,d), A-WS_2_@MWCNTs are apparently rooted within the PUE matrix, which ascertains suitable adhesion between the matrix and the filler. This phenomenon clearly indicates that the pull-out mechanism of the nanocomposites hinders the crack extension and promotes the improvement of the composite toughness and energy absorption capacity.

**Figure 9:**
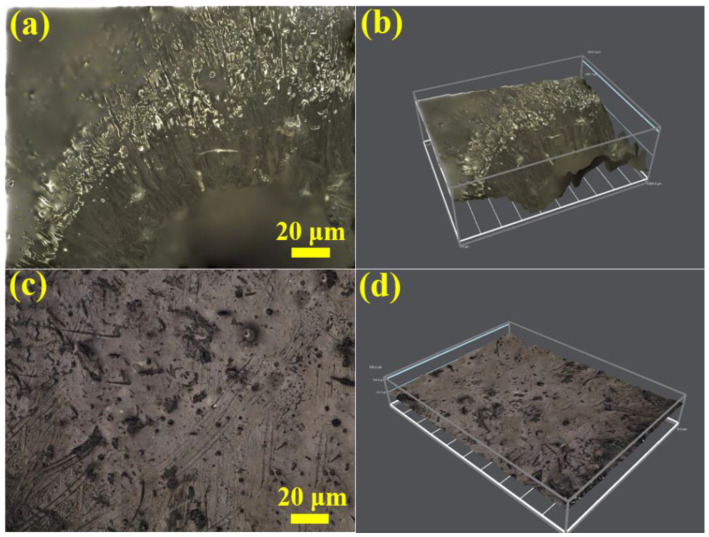
(**a**,**c**) Ultra-depth of field 3D microscopy images of (**a**) pure PUE and (**c**) A-WS_2_@MWCNTs/PUE surface, and (**b**,**d**) 3D topography images corresponding to the (**a**) pure PUE and (**c**) A-WS2@MWCNTs/PUE.

Figure 9a–d shows the surface morphology of pure PUE and 1.5%A-WS_2_@MWCNTs/PUE under the ultra-depth of field three-dimensional microsystem after impact. The center of the pure PUE is severely damaged, and a ring-shaped macroscopic shear band appears around the hole, which indicates that the external force on the PUE extends from the periphery to the center and finally causes the perforation damage at the center. On the contrary, many discontinuous micro-cracks appear on the surface of A-WS_2_@MWCNTs. The micro-cracks stop spreading at the distribution of nanoparticles, reducing the stress concentration in the center and protecting the PUE from damage. This also proves that A-WS_2_@MWCNTs nanofiller has the function of inhibiting crack propagation.

**Figure 10:**
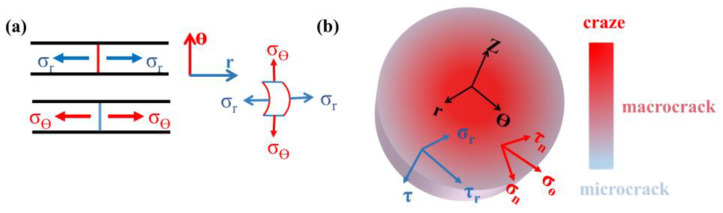
(**a**) Schematic diagram of radial stress and circumferential stress, and (**b**) The schematic diagram of the decomposition of radial and circumferential stresses and the degree of damage to the specimen.

A simple damage model (Figure 10) of PUE under impact is established by observing the microscopic morphology of the specimens and referencing the elliptic criterion (metal glass stamping experiments [40]) and the Mohr–Coulomb (flexible polymer impact loading experiments [41]). This model helps explain the preventing crack propagation role of A-WS_2_@MWCNTs in the impact process. PUE is loaded by radial normal, *σ_r_*, and circumferential normal stresses, *σ_θ_*, under the impact of SHPB. In the stress element of the θ-Z plane, the *σ_θ_* can be decomposed into two-component stresses on any stress plane, namely shear stress, *τ_n_*, and normal stress, *σ_n_*. Under the action of two-component stress, a radial shear band is produced. The motion of radial shear bands can be explained in terms of elliptic criteria:

(5) $$f\left(\tau_{n},\;\sigma_{n}\right)={\left(\tau_{n}/\tau_{0}\right)}^{2}+{\left(\sigma_{n}/\sigma_{0}\right)}^{2},$$

(6) $$f_{max}(\tau_{n},\;\sigma_{n})\geq f_{0}\left(\tau_{0\;},\;\sigma_{0}\right),$$

In elliptic criteria, *τ*_0_ is the critical shear strength; σ_0_ is the critical dissociation strength of PUE, which depends on the material. When the maximum value of the function f (*τ_n_*, *σ_n_*) increases to the critical function value of PUE, the joint action of shear stress *τ_n_* and normal stress *σ_n_* occurs, and the radial shear zone starts to form and propagate along the radial direction. Therefore, according to the ellipse criterion, the radial shear band is driven by circumferential tensile stress, *σ_θ_*.

On the other hand, in the stress element of the R-Z plane, there is a pure shear stress *τ* in the Z direction perpendicular to the R direction. According to $\vec{\tau_{r}}=\vec{\tau }+\vec{\sigma_{r}}$, radial normal stress σ_r_ can be changed to synthetic stress *τ_r_*, according to Mohr–Coulomb criterion:

(7) $$\left|\tau_{r}\right|\geq \tau_{n}=\tau_{0}+\mu \sigma_{n},$$

In Mohr–Coulomb criterion, $\left|\tau_{r}\right|$ is the absolute value of the combined stress of radial normal stress, *τ_r_* is the critical shear fracture strength of the PUE, *τ*_0_ is the critical shear strength of the material, and *μ* is the material constant of PUE. Therefore, when the resultant stress *τ*_r_ is in contact with the critical shear fracture line, the circumferential shear band begins to form and expand in the circumferential direction [40]. Therefore, the synthetic pure shear stress *τ_r_* causes the formation and movement of the circumferential shear band.

In conclusion, the radial shear band is caused by *σ_θ_*, while the circumferential shear band is excited by pure shear stress *τ_r_*. Different deformation mechanisms determine the failure mode of the material, and the formation and movement of the double shear band lead to the final central failure of PUE. The essence of the PUE deformation process is the competition process between *σ_θ_* and *τ_r_*. However, during the deformation of A-WS_2_@MWCNTs/PUE, A-WS_2_@MWCNTs increases the critical shear strength *τ*_0_ and the critical dissociation strength *σ*_0_, which makes radial shear band difficult to form and move. Similarly, the formation and expansion of circumferential shear bands are impeded. Finally, the two shear bands do not extend deep, and some areas form a discontinuous plastic deformation zone, which effectively improves energy absorption. In addition, for the dominant plastic deformation *τ_r_*, A-WS_2_@MWCNTs can effectively prevent the regional damage caused by the accumulation of plastic deformation bands through its own deformation, which is also due to the excellent compatibility and dispersion of A-WS_2_@MWCNTs.

## 4. Conclusions

In this experiment, the A-WS_2_@MWCNTs composite filler was prepared by in situ growth and in situ grafting to improve the impact resistance of PUE. Through a static compression test, dynamic impact test, microscopic morphology observation, and failure mechanism analysis, we can draw the following conclusions:

1. Compared with pure PUE, the static compressive strength and dynamic yield stress of composite PUE are increased by 144.2% and 331.7%, respectively. The addition of composite filler enhances the strength and toughness of PUE and avoids the damage and softening caused by stress concentration and heat concentration under the impact effect. Most notably, the composite PUE remains intact under the successive shocks, while the pure PUE is destroyed by a single impact. The suitable impact resistance of A-WS_2_@MWCNTs/PUE is attributed to the suitable interface interaction between nanoparticles and PUE and the synergy between nanoparticles.
2. According to the surface morphology analysis, polyurethane is prone to perforation damage in the central area under high-speed impact. However, A-WS_2_@MWCNTs/PUE has a large plastic deformation zone, which absorbs external energy, delays the growth of macroscopic cracks, and prevents damage to the specimen. In addition, the morphology of the section shows that A-WS_2_@MWCNTs is rooted in the PUE matrix, indicating that the adhesion between the matrix and the filler is suitable. This indicates that the pull-out mechanism of the nanocomposites impedes the crack propagation and promotes the toughness and energy absorption capacity of the composites.
3. It can be concluded that the deformation process of PUE is a competitive process between *σ_θ_* and *τ_r_* by studying the damage mechanism of PUE. For A-WS_2_@MWCNTs/PUE, A-WS_2_@MWCNTs increases the critical shear strength τ_0_ and the critical dissociation strength *σ*_0_, making it difficult for the radial shear band to form and move. Similarly, the formation and expansion of circumferential shear zones are also hindered. Finally, the two shear bands do not extend deeply, and some regions form a discontinuous plastic deformation zone, which effectively improves energy absorption.
4. Based on the characteristics of A-WS_2_@MWCNTs/PUE with high impact strength, it is worthy of being widely used in the field of impact protection. In addition, the damage formation process of PUE material is analyzed, and the preventing effect of nanofiller on crack propagation is explained, which lays a theoretical foundation for the subsequent development of impact-resistant nanocomposite PUE.

## Figures and Tables

**Table 1:** Formulations of composite PUE and related static compression modulus.

Sample	Relative Mass Ratio A: B: Nanoparticles (g)	Elasticity Modulus (MPa)	Maximum Strength (MPa)	Constant Pressure Intensity (10%) (MPa)
Pure PUE	5:2:0	95.8	90.2	11.3
1.0 wt.% WS_2_@MWCNTs/PUE	5:2:0.070	131.4	137.7	16.0
1.5 wt.% WS_2_@MWCNTs/PUE	5:2:0.105	217.6	233.7	27.6
2.0 wt.% WS_2_@MWCNTs/PUE	5:2:0.140	84.1	77.0	9.9
2.5 wt.% WS_2_@MWCNTs/PUE	5:2:0.175	73.3	71.1	9.0
3.0 wt.% WS_2_@MWCNTs/PUE	5:2:0.210	110	61.5	10.3
1.5 wt.% WS_2_/PUE	5:2:0.105	134.3	176.0	23.3
1.5 wt.% MWCNTs/PUE	5:2:0.105	180.3	206.5	24.1

## Data Availability

Not applicable.

## References

[1] Mostafavi A., Daemi H., Rajabi S., Baharvand H. (2021). Highly tough and ultrafast self-healable dual physically crosslinked sulfated alginate-based polyurethane elastomers for vascular tissue engineering. Carbohydr. Polym..

[2] Wang Z., Zhou J., Liang H., Ye S., Zou J., Yang H. (2020). A novel polyurethane elastomer with super mechanical strength and excellent self-healing performance of wide scratches. Prog. Org. Coat..

[3] Hu F., Gao J., Zhang B., Qi F., Zhao N., Ouyang X. (2021). Effects of Modified Al_2_O_3_-Decorated Ionic Liquid on the Mechanical Properties and Impact Resistance of a Polyurethane Elastomer. Materials.

[4] Zhang G., Yin T., Nian G., Suo Z. (2021). Fatigue-resistant polyurethane elastomer composites. Extreme Mech. Lett..

[5] Zheng Z., Wang Z., Wang L., Liu J., Wu Y., Zhang L. (2016). Dispersion and shear-induced orientation of anisotropic nanoparticle filled polymer nanocomposites: Insights from molecular dynamics simulation. Nanotechnology.

[6] Araby S., Meng Q., Zhang L., Zaman I., Majewski P., Ma J. (2015). Elastomeric composites based on carbon nanomaterials. Nanotechnology.

[7] Fei Y., Chen F., Fang W., Xu L., Ruan S., Liu X., Zhong M., Kuang T. (2020). High-strength, flexible and cycling-stable piezo-resistive polymeric foams derived from thermoplastic polyurethane and multi-wall carbon nanotubes. Compos. Part B Eng..

[8] He Z., Byun J.-H., Zhou G., Park B.-J., Kim T.-H., Lee S.-B., Yi J.-W., Um M.-K., Chou T.-W. (2019). Effect of MWCNT content on the mechanical and strain-sensing performance of Thermoplastic Polyurethane composite fibers. Carbon.

[9] Zhao W., Liu L., Leng J., Liu Y. (2019). Thermo-mechanical behavior prediction of particulate reinforced shape memory polymer composite. Compos. Part B Eng..

[10] Wang E., Dong Y., Islam Z., Yu L., Liu F., Chen S., Qi X., Zhu Y., Fu Y., Xu Z. (2019). Effect of graphene oxide-carbon nanotube hybrid filler on the mechanical property and thermal response speed of shape memory epoxy composites. Compos. Sci. Technol..

[11] Gao Y., Liu J., Shen J., Wu Y., Zhang L. (2014). Influence of various nanoparticle shapes on the interfacial chain mobility: A molecular dynamics simulation. Phys. Chem. Chem. Phys..

[12] Liu J., Shen J., Zheng Z., Wu Y., Zhang L. (2015). Revealing the toughening mechanism of graphene–polymer nanocomposite through molecular dynamics simulation. Nanotechnology.

[13] Sethulekshmi A., Jayan J.S., Saritha A., Joseph K. (2021). Insights into the reinforcibility and multifarious role of WS2 in polymer matrix. J. Alloy. Compd..

[14] Houssat M., Villeneuve-Faure C., Dignat N.L., Cambronne J.-P. (2021). Nanoscale mechanical and electrical characterization of the interphase in polyimide/silicon nitride nanocomposites. Nanotechnology.

[15] Chen D., Tiwari S.K., Ma Z.Y. (2015). Phase Behavior and Thermo-Mechanical Properties of IF-WS2 Reinforced PP–PET Blend-Based Nanocomposites. Polymers.

[16] Divya G., Suresha B. (2021). Impact of nano-silicon dioxide on mechanical properties of carbon fabric reinforced epoxy composites. Mater. Today Proc..

[17] Kim K., Ju H., Kim J. (2016). Surface modification of BN/Fe3O4 hybrid particle to enhance interfacial affinity for high thermal conductive material. Polymer.

[18] Pan Z., Xiong J., Liang S., Zou M. (2017). Transient deformation and heat generation of solid polyurethane under impact compression. Polym. Test..

[19] Fan J., Weerheijm J., Sluys B. (2016). Compressive response of a glass–polymer system at various strain rates. Mech. Mater..

[20] Pankow M., Attard C., Waas A.M. (2009). Specimen size and shape effect in split Hopkinson pressure bar testing. J. Strain Anal. Eng. Des..

[21] Sarva S.S., Deschanel S., Boyce M.C., Chen W. (2007). Stress–strain behavior of a polyurea and a polyurethane from low to high strain rates. Polymer.

[22] Chen W., Lu F., Cheng M. (2002). Tension and compression tests of two polymers under quasi-static and dynamic loading. Polym. Test..

[23] Chen W., Zhang B., Forrestal M.J. (1999). A split Hopkinson bar technique for low-impedance materials. Exp. Mech..

[24] Sharifi T., Nitze F., Barzegar H.R., Tai C.-W., Mazurkiewicz-Pawlicka M., Małolepszy A., Stobinski L., Wagberg T. (2012). Nitrogen doped multi walled carbon nanotubes produced by CVD-correlating XPS and Raman spectroscopy for the study of nitrogen inclusion. Carbon.

[25] Panahi-Sarmad M., Chehrazi E., Noroozi M., Raef M., Razzaghi-Kashani M., Baian M.A.H. (2019). Tuning the Surface Chemistry of Graphene Oxide for Enhanced Dielectric and Actuated Performance of Silicone Rubber Composites. ACS Appl. Electron. Mater..

[26] Kumar S., Gupta T.K., Varadarajan K. (2019). Strong, stretchable and ultrasensitive MWCNT/TPU nanocomposites for piezoresistive strain sensing. Compos. Part B Eng..

[27] Ferdous S.F., Sarker F., Adnan A. (2013). Role of nanoparticle dispersion and filler-matrix interface on the matrix dominated failure of rigid C60-PE nanocomposites: A molecular dynamics simulation study. Polymer.

[28] Balazs A., Emrick T., Russell T.P. (2006). Nanoparticle Polymer Composites: Where Two Small Worlds Meet. Science.

[29] Xia C., Li J., Cao Y., Kou B., Xiao X., Fezzaa K., Xiao T., Wang Y. (2015). The structural origin of the hard-sphere glass transition in granular packing. Nat. Commun..

[30] Zhang P., Wang Z., Zhao P., Zhang L., Jin X., Xu Y. (2019). Experimental investigation on ballistic resistance of polyurea coated steel plates subjected to fragment impact. Thin-Walled Struct..

[31] Zhou R.X., Hu H., Chen N.L., Feng X.W. (2005). An Experimental and Numerical Study on the Impact Energy Absorption Characteristics of the Multiaxial Warp Knitted (MWK). Reinforced. Compos. J. Compos. Mater..

[32] Shaker K., Jabbar A., Karahan M., Karahan N., Nawab Y. (2016). Study of dynamic compressive behaviour of aramid and ultrahigh molecular weight polyethylene composites using Split Hopkinson Pressure Bar. J. Compos. Mater..

[33] Stachurski Z. (1997). Deformation mechanisms and yield strength in amorphous polymers. Prog. Polym. Sci..

[34] Richeton J., Ahzi S., Vecchio K., Jiang F., Adharapurapu R. (2006). Influence of temperature and strain rate on the mechanical behavior of three amorphous polymers: Characterization and modeling of the compressive yield stress. Int. J. Solids Struct..

[35] Fan J., Weerheijm J., Sluys B. (2015). Glass interface effect on high-strain-rate tensile response of a soft polyurethane elastomeric polymer material. Compos. Sci. Technol..

[36] Ackland K., Anderson C., Ngo T. (2013). Deformation of polyurea-coated steel plates under localised blast loading. Int. J. Impact Eng..

[37] Roland C., Twigg J., Vu Y., Mott P. (2007). High strain rate mechanical behavior of polyurea. Polymer.

[38] Zhang L., Yao X., Zang S., Gu Y. (2015). Temperature- and strain rate-dependent constitutive modeling of the large deformation behavior of a transparent polyurethane interlayer. Polym. Eng. Sci..

[39] Mousa M., Dong Y. (2018). Novel three-dimensional interphase characterisation of polymer nanocomposites using nanoscaled topography. Nanotechnology.

[40] Fan J., Fu T. (2012). Deformation and fracture mechanism of materials under the punch loading. Mater. Lett..

[41] Fan J., Weerheijm J., Sluys B. (2018). Deformation to fracture evolution of a flexible polymer under split Hopkinson pressure bar loading. Polym. Test..

